# Regulation of Autophagic Signaling by Mechanical Loading and Inflammation in Human PDL Fibroblasts

**DOI:** 10.3390/ijms21249446

**Published:** 2020-12-11

**Authors:** Kim Blawat, Alexandra Mayr, Miriam Hardt, Christian Kirschneck, Marjan Nokhbehsaim, Christian Behl, James Deschner, Andreas Jäger, Svenja Memmert

**Affiliations:** 1 Center of Dento-Maxillo-Facial Medicine, Department of Orthodontics, University of Bonn Medical Center, 53111 Bonn, Germany; kim.blawat@ukbonn.de (K.B.); alexandra.mayr@ukbonn.de (A.M.); miriam@dicktenweb.de (M.H.); andreas.jaeger@ukbonn.de (A.J.); 2 Department of Orthodontics, University Hospital Regensburg, 93042 Regensburg, Germany; Christian.Kirschneck@klinik.uni-regensburg.de; 3 Center of Dento-Maxillo-Facial Medicine, Section of Experimental Dento-Maxillo-Facial Medicine, University of Bonn Medical Center, 53111 Bonn, Germany; m.saim@uni-bonn.de; 4 The Autophagy Lab, Institute of Pathobiochemistry, University Medical Center of the Johannes Gutenberg University, 55099 Mainz, Germany; cbehl@uni-mainz.de; 5 Department of Periodontology and Operative Dentistry, University Medical Center of the Johannes Gutenberg University, 55131 Mainz, Germany; james.deschner@uni-mainz.de

**Keywords:** autophagy, mammalian target of rapamycin (mTOR) signaling pathway, inflammatory conditions, mechanical stress, orthodontic tooth movement

## Abstract

Autophagy (cellular self-consumption) is a crucial adaptation mechanism during cellular stress conditions. This study aimed to examine how this important process is regulated in human periodontal ligament (PDL) fibroblasts by mechanical and inflammatory stress conditions and whether the mammalian target of rapamycin (mTOR) signaling pathway is involved. Autophagy was quantified by flow cytometry. Qualitative protein phosphorylation profiling of the mTOR pathway was carried out. Effects of mTOR regulation were assessed by quantification of important synthesis product collagen 1, cell proliferation and cell death with real-time PCR and flow cytometry. Autophagy as a response to mechanical or inflammatory treatment in PDL fibroblasts was dose and time dependent. In general, autophagy was induced by stress stimulation. Phosphorylation analysis of mTOR showed regulatory influences of mechanical and inflammatory stimulation on crucial target proteins. Regulation of mTOR was also detectable via changes in protein synthesis and cell proliferation. Physiological pressure had cell-protective effects (*p* = 0.025), whereas overload increased cell death (*p* = 0.003), which was also promoted in long-term inflammatory treatment (*p* < 0.001). Our data provide novel insights about autophagy regulation by mechanical and inflammatory stress conditions in human PDL fibroblasts. Our results suggest some involvement of the mTOR pathway in autophagy and cell fate regulation under the named conditions.

## 1. Introduction

Orthodontic tooth movement, as a therapeutic action to correct misaligned and mispositioned teeth, is not inducible without exposing the structural components of the periodontium, consisting of gingiva, periodontal ligament (PDL), root cementum and alveolar bone, to various types of stress. Due to its position and function as the central element of connection between tooth and bone, the PDL, and in particular its dominant cell type, PDL fibroblasts, play a key role in adaptation to stress [1]. PDL fibroblasts are derived from ectomesenchyme cranial neural crest cells. They have unique properties compared to other types of fibroblasts, as they are able to differentiate into cementoblasts and osteoblasts [2]. PDL fibroblasts are responsible for tissue remodeling and the synthesis of extracellular matrix components, such as elastin, glycoproteins and glycosaminoglycans, but mainly fibrillar type I collagen [3,4,5].

Orthodontic tooth movement is induced by the application of mechanical stress on the periodontium, which leads to regional biomechanical strain on cells and tissues, which is followed by subsequent sterile inflammation involving the release of inflammatory cytokines, predominantly interleukin-1 (IL-1β), by the affected tissues. Whereas moderate pressure is important for periodontal tissue homeostasis, mechanical overload on the periodontium is followed by cell damage associated with an increased cell death rate in PDL fibroblasts and dysregulated bone remodeling [6,7]. Furthermore, a small amount of IL-1β has been shown to be beneficial for periodontal remodeling, including wound healing, whereas IL-1β overproduction has been demonstrated to play a role in periodontal disease etiologies [8]. To analyze tooth movement in vitro, established models exist where PDL cells are treated with comparable pressure applications or physiological concentrations of IL-1β [1,9,10,11,12].

Autophagy, the physiological process of self-consumption, is an essential mechanism for stress adaptation [13,14]. Under stress conditions, autophagy is involved in the determination of cell fate; it can ensure cell survival, but also induce cell death [15]. Three types of autophagy are described in eukaryotic cells: macroautophagy, microautophagy and chaperone-mediated autophagy. Macroautophagy is characterized by the fusion of membrane-bound vesicles (autophagosomes) with lysosomes to form autolysosomes, where the degradation of misfolded proteins, non-functional organelles and intracellular pathogens takes place [16]. Hereafter “autophagy” is used as a synonym for “macroautophagy”. The process of autophagy comprises four phases: initiation, elongation (formation of the autophagosome), maturation and degradation [17]. In previous studies we were able to show the importance of autophagy in periodontal stress regulation. Autophagy was induced by tensile strain in PDL fibroblasts in a dose-dependent manner [18,19]. Additionally, a recent study suggests that autophagy is a crucial process for pressure regulation in PDL fibroblasts [20]. Furthermore, the inflammatory cytokine IL-1β was shown to regulate autophagy-associated genes in a dose-dependent manner as well, suggesting an important role of autophagy in periodontal inflammation [11,12].

Autophagy is regulated by a plethora of signaling pathways. One of the most crucial is the mammalian target of rapamycin (mTOR) signaling pathway, which inhibits autophagy [21]. Besides autophagy, this essential pathway for cell fate determination regulates cell growth, proliferation, differentiation and protein synthesis [15,21]. Upstream, phosphoinositide 3-kinase/-serine/threonine protein kinase Akt (PI3K/AKT) is an important regulating pathway of mTOR. PI3K activates AKT via phosphorylation, and furthermore, AKT can have anabolic effects on the mTOR complex, which promotes transcription of downstream proteins [21]. One of the main inhibitors of the PI3K/AKT pathway is phosphatase and tensin homolog deleted on chromosome 10 (PTEN). PTEN is able to limit the activity of AKT [22]. Downstream, mTOR interacts among others with eukaryotic translation initiation factor 4E (eIF4E)-binding protein 1 (4E-BP1) and 70-kDa ribosomal protein S6 kinase (P70S6K) to promote protein synthesis [23].

Autophagy is highly sensitive to changes in mechanical pressure. It has already been discovered by King et al. that autophagy correlates with the extent of pressure. Their results suggest that induction of autophagy by pressure might be mTOR-independent, whereas other stimuli such as starvation induce autophagy induction via mTOR [24]. In response to a lack of nutrients or growth factors, an inhibition of mTOR leads to the initiation of autophagy. Furthermore, the widely-known autophagy-inducer rapamycin targets mTOR [25]. The intracellular signaling pathway mTOR critically controls a wide range of cellular functions and is an important pathway for the determination of cell fate. Activating this signal pathway controls protein synthesis and promotes cell proliferation, cell growth and cell differentiation [14,26].

Therefore, the aim of this study was to investigate how mechanical and inflammatory stresses of different magnitudes influence autophagy activity in PDL fibroblasts. A further objective was to analyze whether the mTOR pathway is affected by different magnitudes of pressure or inflammatory stimulation. We hypothesize that cell fate as an effect of mTOR and autophagy regulation is also influenced by mechanical pressure or by inflammatory conditions.

## 2. Results

### 2.1. Effects of Mechanical and Inflammatory Stimulation on Autophagy

**Figure 1:**
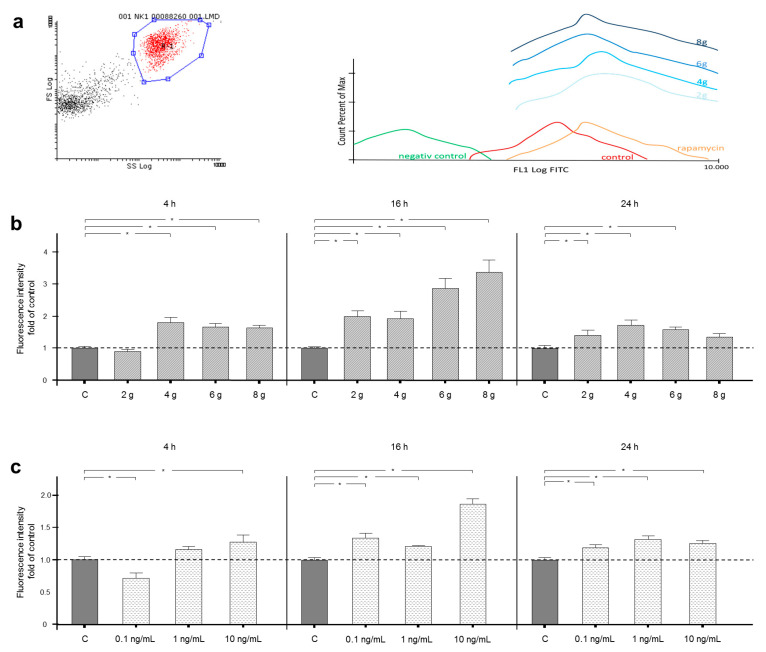
(**a**) For FACS analysis, the decisive cell population was defined and gated, as shown in the left panel. As pictured by the circled gate R1, the further studied cell population was selected by forward (FS) and side scatter (SS), which indicate size and granulation of the particles (left panel). In addition to the mechanically stimulated groups, the analysis of 3 defined control groups (negative control—undyed; unstimulated control; and positive control—pharmacologically induced autophagy) were carried out. Fluorescence peaks are shown along the x-axis to visualize the amount of fluorescence in the FL1 log FITC channel. The more to the right the peaks are positioned on the x-axis, the more fluorescence was measured in the FITC channel. On the y-axis, the percentages of the maximum fluorescence events are shown (right panel). (**b**) Effects on autophagosome accumulation, indicated by fluorescence, after 4, 16 or 24 h of treatment with 2, 4, 6 or 8 g/cm^2^ or no pressure. Autophagic flux was interrupted by chloroquine treatment. (**c**) Effects on autophagosome accumulation, indicated by fluorescence, after 4, 16 or 24 h of treatment with 0.1, 1.0 or 10 ng/mL IL-1β or no treatment. Autophagic flux was interrupted by chloroquine treatment. Fluorescence intensity is shown as fold of control. Mean ± SEM (*n* = 12 replicates); * significant (*p* < 0.05) difference between groups.

Autophagosome accumulation was measured by FACS analysis after mechanical and inflammatory stimulation of PDL fibroblasts for 4, 16 or 24 h (Figure 1a–c).

For positive controls, autophagy was induced by rapamycin, whereas unstained cells served as negative controls (Figure 1a).

Mechanical pressure of 4, 6 and 8 g/cm^2^ for 4 h led to a significant nearly 2-fold increase in fluorescence intensity, whereas 2 g/cm^2^ did not change fluorescence intensity significantly in the same timeframe. After 16 h of mechanical loading, the fluorescence intensity was significantly increased by all tested pressure levels compared to control cells. The fluorescence intensity was increased more than 3-fold in the 8 g/cm^2^ group. However, after 24 h, application of 8 g/cm^2^, the largest magnitude of pressure tested, did not enhance fluorescence intensity anymore. PDL fibroblasts treated with the lower magnitudes of 2, 4 and 6 g/cm^2^ still showed significantly increased fluorescence intensity compared to control after 24 h stimulation (Figure 1b).

Interestingly, inflammatory stimulation with 0.1 ng/mL IL-1β for 4 h decreased fluorescence intensity significantly by over 25%, whereas a concentration of 1 ng/mL did not have any significant effects. However, a concentration of 10 ng/mL IL-1β increased fluorescence intensity by over 25% in the same timeframe. When PDL fibroblasts were cultured under inflammatory conditions for 16 and 24 h, all assessed concentrations of IL-1β raised fluorescence intensity significantly (Figure 1c).

### 2.2. Influences of Mechanical Force Application and Inflammation on the mTOR Signaling Pathway

The human mTOR Signaling Phospho Specific Antibody Array determines site-specific phosphorylation changes of upstream and downstream proteins of the mTOR pathway.

Overall, ratios shifted more towards the phosphorylated than the non-phosphorylated proteins due to mechanical or inflammatory stimulation (Table 1). Pressure stimulation of 2 g/cm^2^ representing physiological conditions during orthodontic tooth movement induced lesser ratio changes than stress conditions, i.e., overload and inflammation.

Upstream, the balance of non-phosphorylated and phosphorylated PI3K shifted towards phosphorylation following overloading or inflammation of 1.7 and 1.6-fold respectively (PI3-kinase p85-subunit alpha/gamma). The ratio of PTEN also shifted in favor of phosphorylation underloading, overloading and inflammatory conditions up to 2-fold (Table 1).

The balance of AKT phosphorylation shifted towards decreased phosphorylation by physiological loading conditions to 0.7-fold. Overload increased the ratio of phosphorylated AKT1 at phosphorylation site Ser124, but had an adverse effect on phosphorylated AKT1 at phosphorylation site Tyr474. Inflammation elevated the ratio of phosphorylated AKT1 at phosphorylation sites AB-124 and AB-450 by more than 1.5-fold (Table 1). However, fold changes of phosphorylated and non-phosphorylated protein AKT showed an overall downregulation in comparison to controls (Table 2). Fold changes of the mTOR protein also suggested a downregulation of less than 0.75-fold in comparison to controls (Table 2).

Downstream, multiple proteins such as 4E-BP1 and P70S6K were assessed at different binding sites. Pressure of 2 g/cm^2^ led to a decrease of phosphorylated 4E-BP1 at phosphorylation site Thr45, whereas IL-1β increased the ratio of phosphorylated 4E-BP1. The balance of phosphorylated versus non-phosphorylated P70S6K was shifted to the phosphorylated protein by all stress stimulations tested (Table 1). Fold changes of phosphorylated P70S6K at phosphorylation site Ser418 were in all treatment groups more than 1.6 increased (Table 2).

Further ratio changes can be found in Table 1, whereas all fold changes assessed can be viewed in Supplementary Materials (Table S1).

### 2.3. Regulation of Collagen 1 Gene Expression by Mechanical and Inflammatory Stimulation

**Figure 2:**
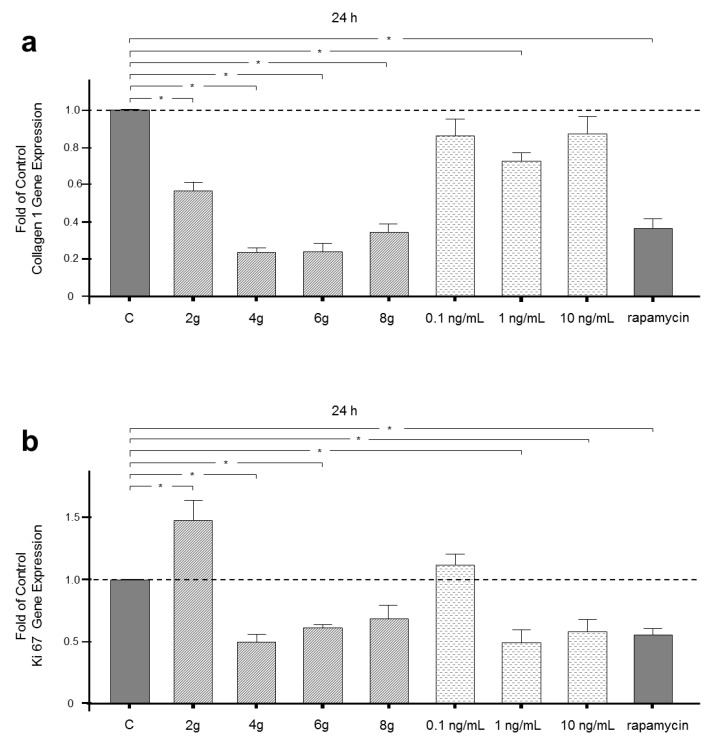
(**a**) Effects of 2, 4, 6 or 8 g/cm^2^ pressure or 0.1, 1.0 or 10 ng/mL IL-1β on collagen I gene expression after 24 h. Untreated cells served as controls. Rapamycin treatment inhibited mTORC1 as the negative control. (**b**) Effects of 2, 4, 6 or 8 g/cm^2^ pressure or 0.1, 1.0 or 10 ng/mL IL-1β on Ki67 gene expression after 24 h. Untreated cells served as controls. Rapamycin treatment inhibited mTORC1 as the negative control. Mean ± SEM (*n* = 9 replicates); * significant (*p* < 0.05) difference between groups.

Activation of mTOR controls protein synthesis. Therefore, gene expression of collagen 1, which is the most important component of extracellular matrix produced by PDL fibroblasts, was assessed after 24 h of stimulation. Collagen 1 gene expression was significantly reduced by inhibition of mTOR following rapamycin treatment. Moreover, all magnitudes of mechanical pressure decreased gene expression of collagen 1 significantly. A reduction of more than 75% was found in the 4 and 6 g/cm^2^ group. Inflammatory stimulation with an intermediate concentration of IL-1β also led to a reduction in the gene expression of collagen 1 by more than 50%. Low and high concentrations of IL-1β treatment, however, did not have a significant influence on collagen 1 gene expression (Figure 2a).

### 2.4. Influence of Mechanical and Inflammatory Stimulation on Gene Expression of Proliferation Marker Ki67

The cell fate pathway mTOR regulates cell proliferation. Therefore, gene expression of the specific marker of cell proliferation Ki67 was analyzed after mechanical and inflammatory stimulation. Inhibition of mTORC1 by rapamycin led to a reduction in Ki67 gene expression. A physiological pressure of 2 g/cm^2^ had adverse effects on Ki67 gene expression and induced an upregulation of Ki67 nearly up to 50%. Higher pressure magnitudes of 4 and 6 g/cm^2^ downregulated Ki67 gene expression. Interestingly, pressure of 8 g/cm^2^ had no significant effect of Ki67 gene expression (Figure 2b). Low concentration IL-1β treatment did not change Ki67 gene expression, whereas higher concentrations of 1 and 10 ng/mL IL-1β reduced Ki67 gene expression significantly by more than 40% (Figure 2b).

### 2.5. Effects of Mechanical and Inflammatory Stimulation on Cell Death

Cell death is determined by the mTOR pathway in a close interplay with autophagy. Cells positive for propidium iodide (PI) were identified by FACS analysis.

**Figure 3:**
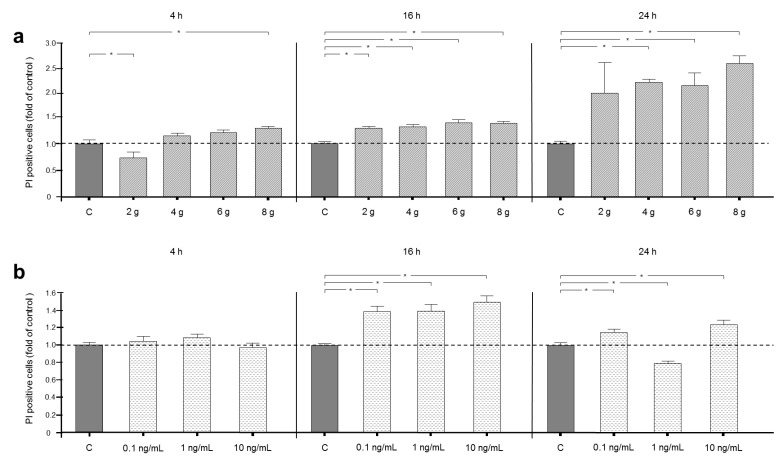
(**a**) Effects on cell death, indicated by fluorescence, after 4, 16 or 24 h of treatment with 2, 4, 6 or 8 g/cm^2^ or no pressure without chloroquine. (**b**) Effects on cell death, indicated by fluorescence, after for 4, 16 or 24 h of treatment with 0.1, 1.0 or 10 ng/mL IL-1β or no treatment without chloroquine. Fluorescence intensity is shown as fold of control. Mean ± SEM (*n* = 9 replicates); * significant (*p* < 0.05) difference between groups.

Interestingly, an application of 2 g/cm^2^ for 4 h significantly reduced cell death by over 20% and consequently had a cell-protective effect. The same amount of pressure significantly increased PI-positive cells after 16 h. Interestingly, 24 h of treatment with 2 g/cm^2^ did not change the number of PI-positive cells significantly in comparison to controls. After 4 h of pressure application, only high pressure treatment of 8 g/cm^2^ induced cell death by over 20% significantly, whereas 4 and 6 g/cm^2^ had no significant effect on the number of dead cells. However, after 16 and 24 h of pressure with 4, 6 and 8 g/cm^2^ increased the number of dead cells significantly—more than 2-fold increases (Figure 3a).

Inflammatory stimulation for 4 h did not change the cell death rate. Cell culture under inflammatory conditions for 16 h led to an increase in dead cells by over 25% in comparison to controls. Interestingly, after 24 h, the low concentration and high concentration of IL-1β raised cell death numbers, whereas an intermediate concentration of IL-1β reduced the number of dead cells in comparison to controls (Figure 3b).

## 3. Discussion

Our results suggest the dose and time-dependency of autophagic activity as consequences of mechanical and inflammatory stimulation. In general, autophagic flux was induced by stress stimulation, whereas a low pressure or low concentration inflammation treatment applied over a short timeframe did not influence or even reduce autophagy, respectively. Phosphorylation analysis of a key upstream autophagy regulatory pathway, mTOR, suggested the impacts of mechanical and inflammatory stimulation via the PI3K/AKT signaling pathway. Downstream targets of mTOR, such as 4EPB1 and p70S6K, were also regulated by mechanical and inflammatory stimulation. Furthermore, mechanical and inflammatory stimulation had downregulating effects on collagen1 gene expression, similarly to mTOR inhibition. Analysis of proliferation marker Ki67 revealed reduced gene expression after mTOR inhibition and after stress stimulation. However, a physiological pressure of 2 g/cm^2^ or a low concentration of inflammatory IL-1β increased or did not change gene expression, respectively. Moreover, a physiological pressure of 2 g/cm^2^ reduced cell death. Overload, conversely, increased the number of dead cells. Additionally, cell death was not elevated by short-term IL-1β treatment, but promoted by long-term treatment.

In the course of orthodontic tooth movement, an aseptic inflammatory reaction is induced in the periodontium, which causes the release of pro-inflammatory mediators, such as IL-1β, by local cells. Due to their location, PDL fibroblasts play an important role in the periodontium during adaptation to mechanical and inflammatory stress [1,11,12]. To gain knowledge about adaptation mechanisms on the cellular level, we used established in vitro models for mechanical and inflammatory stress [9,11,12]. For the mechanical stimulation we used different magnitudes of stress. The amount of 2 g/cm^2^ has been established as physiological pressure by multiple studies and was also used in our experimental design [10,27,28]. Cells treated with a pressure of 4 g/cm^2^ have been described to suffer cell damage as a result of excessive pressure [10]. However, in our experiments we found cell death to be induced only after treatment with 8 g/cm^2^ for a short application period of 4 h. However, after long-term pressure treatment of 24 h, cell death was significantly induced by a pressure of 4 g/cm^2^.

In vitro models provide us with the opportunity for a differentiated view on stress stimulation. This is particularly relevant for autophagy analysis, as autophagy can be induced by multiple types of cellular stress [13]. However, we should bear in mind that an in vitro model can never achieve the complexity of the in vivo situation. Therefore, the aim of future studies is to apply our knowledge in an in vivo model.

Autophagy is an important mechanism for cellular and functional homeostasis in PDL fibroblasts [18]. Our findings showed dose and time-dependent activity of this important survival mechanism in PDL fibroblasts after pressure and inflammatory challenges. Earlier studies found dose dependency for autophagy and autophagy-associated genes as a response to tensile strain in PDL fibroblasts [18,19]. Therefore, our findings are consistent with results of previous experiments. An influence of mechanical pressure on autophagy activity was already shown by King and coworkers [24]. A recent study by Chen and colleagues reported autophagy markers in PDL fibroblasts after pressure stimulation [20]. They found these markers to be induced by pressure changes in PDL fibroblasts. Our results complete and expand the findings of this study, since our findings were based on direct quantification of autophagosomes, key vesicular components of the autophagic pathway, whereas Chen and coworkers focused on the induction of important autophagy-associated genes and proteins. Moreover, due to our experimental design, we were able to separate autophagy induction by mechanical stimulation and by hypoxia. Hypoxia is another potent autophagy stimulant, which could also play a role when cells are covered by weights without another option of oxygen supply [13]. The separation of stress stimuli could be accountable for differences between our findings and the results presented by Chen and coworkers. They found a decrease in autophagy induction after 24 h of mechanical stimulation, whereas autophagy in our experiments was still upregulated by pressure stimulation at that time. However, pressure stimulation of 8 g/cm^2^ for 24 h did not increase autophagy. This could be due to the exceedance of a critical stress threshold. When a critical stress threshold is surpassed, autophagy is downregulated by cell death mechanisms [15].

Pro-inflammatory cytokines, such as IL-1β, are accompanied by autophagy, whereas anti-inflammatory cytokines, such as IL-4, IL-10 and IL-13, exerted autophagy-suppressive functions [29]. The dose and time-dependency of important autophagy-associated genes and proteins that respond to inflammatory stimulation have been illustrated in earlier studies [11,12]. Interestingly, our current findings indicate a downregulation of autophagy after 4 h of low-dose IL-1β treatment. The anti-inflammatory effects of low doses of IL-1β have long been recognized [30].

We know from literature that autophagy is a process controlled by rapid phosphorylation of proteins [31]. Therefore, we decided to perform a phospho-specific mTOR antibody array after 4 h, even if autophagy induction does not seem to be upregulated that strongly at this time point, but this could be due to chloroquine treatment.

The critical upstream regulator and molecular switch mTOR regulates a plethora of cellular processes. It is important for cell growth, proliferation, differentiation, protein synthesis and autophagy [21,26,32]. However, whether autophagy by mechanical stimulation is induced via mTOR or is mTOR-independent is a controversial issue [24,26].

In mammalian cells, mTOR occurs in two complexes, mTORC1 and mTORC2 [32,33]. The first complex interacts with 4E-BP1 and S6K1 and promotes protein synthesis. Furthermore, mTORC1 induces growth and inhibits autophagy. This complex is sensitive to inhibition by rapamycin and induces autophagy in its presence. The second complex mTORC2 is only sensitive to rapamycin after a prolonged exposure and mainly regulates cell survival and cytoskeletal organization [21].

The PI3K/AKT signaling pathway is an important upstream regulator of mTOR. Our results suggest at least a partial influence of mTOR on autophagy regulation via the PI3K/AKT signal pathway. These results mirror our findings regarding autophagy activity, which was upregulated due to mechanical and inflammatory stimulation. Other findings also indicate an influence of mechanical stimulation on the PI3K/AKT pathway, depending on the level of mechanical stress [34,35]. King and colleagues did not find a regulatory effect of mechanical stimulation on the AKT pathway [24]. However, these authors did only examine phosphorylation site Ser473 for AKT, which they found to be unchanged by mechanical loading. Our results agree with King and coworkers, as we also found the phosphorylation site Ser473 to be unchanged by mechanical loading. However, other phosphorylation sites of AKT, such as Thr308 or Thr474, were influenced by mechanical loading.

Moreover, protein synthesis is influenced by mTOR via phosphorylation of 4E-BP at several sites. Phosphorylation enables the formation of the protein translation initiation complex [23]. Our experiments revealed that stimulation with a high concentration of IL-1β led to a shift of balance to the phosphorylated protein, which promotes the translation process. Recent findings regarding 4E-BP phosphorylation due to inflammatory stimulation and anti-inflammatory interferon treatment in macrophages showed that the anti-inflammatory treatment dampened the phosphorylation of 4E-BP due to inflammatory stimulation [36]. These results are in line with our data. To our knowledge our study is the first one to link a change in 4E-BP phosphorylation to mechanical stimulation.

Another crucial branch of the mTORC1 pathway is protein and lipid synthesis via P70S6K. P70S6K also enhances and controls cell size [21]. Phosphorylation of P70S6K stimulates the initiation of protein biosynthesis by activating the ribosomal protein S6 [23,37]. Interestingly, all stimulated groups in our experimental design shifted the balance between phosphorylated and non-phosphorylated P70S6K to the phosphorylated kinase in comparison to controls. The effect of mechanical stimulation on P70S6K phosphorylation has already been described in the literature [26,38]. Inflammatory stimulation also activates the S6K pathway, which was shown in murine macrophages [39]. Therefore, data from different studies support our findings regarding P70S6K activation after stimulation with different types of cellular stress. However, King and colleagues did not find changes of P70S6K phosphorylation after mechanical stimulation [24]. However, only phosphorylation site Thr421 of P70S6K was analyzed, which was also not affected by mechanical stimulation according to the results of our study. Instead, phosphorylation sites such as Ser418 and Thr389 were found to be regulated.

As mTOR controls protein synthesis, gene expression of collagen 1 was assessed. However, the reader should bear in mind that gene expression is shown, which can be assumed to lead to protein synthesis. Our results suggest that stress stimulation changes collagen 1 production similarly to inhibition of mTORC1 with rapamycin. These findings coincide with the decreased fold-change of phosphorylated proteins mTOR and 4E-BP. Increased phosphorylation of P70S6K opposes the reduction of collagen 1 gene expression. These results suggest a shift in the composition of synthesized proteins. With regard to the primary function of PDL fibroblasts, mTOR regulation and protein synthesis under stress conditions are particularly interesting and should be investigated in further studies.

Cell proliferation was assessed via gene expression of the proliferation marker Ki67. As expected, mTORC1 inhibition by rapamycin treatment led to a decrease in Ki67 gene expression. Low pressure treatment of 2 g/cm^2^ led to an upregulation of proliferation marker Ki67. These results comply with our apoptosis measurements, as we found cell-protective effects under physiological load. Higher pressure magnitudes resulted in reduced cell proliferation of PDL fibroblasts. Overload of 8 g/cm^2^ had no significant effect on Ki67 gene expression, which could be due to the increased number of dead cells. Low concentration IL-1β treatment did not change Ki67 gene expression, whereas higher concentrations reduced Ki67 gene expression. With regard to low concentration stimulation and pressure application, these results are confirmed by literature [30,40].

The functional dialogue between autophagy, as a pro-survival process, and programmed cell death is part of the physiological regulation of cell turnover in multicellular organisms [41]. Interestingly, many conditions which lead to cell death, also trigger autophagy. Often, both processes occur in a specific sequence. Stress often elicits an autophagic response, especially when it is not lethal. Apoptosis and other cell death mechanisms are activated if stress exceeds a critical threshold in terms of either magnitude or duration. Therefore, autophagy often precedes cell death [15]. When cell death mechanisms are triggered, autophagy can be inactivated. On the other hand, induction of autophagy can lead to an inhibition of cell death [15]. Like autophagy, cell death induction was dependent on the dose and duration of the applied stimulation. Mechanical overload of 8 g/cm^2^ increased cell death at all time points. This provides further evidence that stimulation with 8 g/cm^2^ exceeds a critical threshold and is rightfully characterized as overload. A pressure of 2 g/cm^2^ on the other hand, seemed to have cell-protective effects. These findings are consistent with results of previous experiments, in which cell-protective effects were induced by low tensile strain, whereas high tensile strain application increased cell death [18]. Cell-protective signaling pathways have already been shown to be induced by mechanical stimulation in PDL fibroblasts, which suggested biomechanical stimulation to be important for PDL fibroblast homeostasis [18]. In a previous study PDL fibroblasts were subjected to low magnitudes of biomechanical stress, and subsequently gene expression of anti-apoptotic pathways was induced [42]. A physiological force is apparently required to maintain or even promote cell homeostasis, as already shown in osteoblasts [43]. However, other research groups found autophagy and cell death induction by mechanical stress, which according to our study indicates overload [44,45].

Interestingly, in an inflammatory setting, treatment duration seems to be more important than cytokine concentration in cell death induction. It has already been demonstrated that prolonged treatment with IL-1β at the concentrations of 0.1, 1 or 10 ng/mL induces cell death in tumor cells [46]. However, cell-protective effects of IL-1β have also been shown in immune cells [47]. In previous studies we found damage-regulated autophagy modulator 1 to be a crucial element of p53-mediated apoptosis and autophagy regulation to be induced by inflammatory stimulation in a dose and time-dependent manner, supporting the results of our current study [12].

Taken together, our findings provide novel insights into autophagy activity after mechanical and inflammatory stimulation in PDL fibroblasts. The autophagic response to stress stimulation was dose and time dependent. In general, autophagy was induced by stress stimulation, whereas a brief application of low pressure or low IL-1β concentration did not influence or reduce autophagy. Furthermore, the mTOR pathway was affected by physiological pressure, overload and inflammatory stimulation. Moreover, our results suggest an influence of the mTOR signaling pathway on autophagy activity after mechanical and inflammatory stress. The effects of mechanical and inflammatory stimulation were also detectable via cell fate changes, as analyzed by protein synthesis, proliferation and cell death. Cell death was found to be regulated by mechanical pressure and inflammatory stimulation. Interestingly, a physiological pressure of 2 g/cm^2^ could even exert protective effects, whereas overload increased the number of dead cells. Regarding cell death induction by inflammatory stimulation, treatment duration seems to be more important than IL-1β concentration.

## 4. Materials and Methods

### 4.1. Cell Culture Process

Human PDL fibroblasts (Lonza, Basel, Switzerland) were incubated in Dulbecco’s minimal essential medium (DMEM, Invitrogen, Karlsruhe, Germany), supplemented with 10% fetal bovine serum (FBS, Invitrogen) and 100 units/mL penicillin and 100 μg/mL streptomycin (Invitrogen) in 37 °C humidified atmosphere of 5% CO_2_. Cells were cryopreserved and used for experiments from 4th to 6th passage. Cells (100,000 cells/well) were seeded on lumox^®^ gas-permeable cell culture plates (Sarstedt, Nümbrecht, Deutschland) and grown to 80–90% confluence. For better cell adhesion and growth enhancement on the lumox^®^ surface an attachment factor was used (Biologics Attachment Factor 1X, Gibco, Thermo Fisher Scientific, Waltham, Massachusetts, MA, USA). The FBS concentration was reduced to 1% one day prior to cell stimulation.

### 4.2. Cell Stimulation

PDL fibroblasts were mechanically stimulated with different magnitudes of pressure. Established protocols to apply a compressive force on PDL fibroblasts were used in accordance with earlier studies, which have examined realistic magnitudes of pressure magnitudes in orthodontic treatment considering load and overload for PDL fibroblasts. In brief pressure was applied by glass plates in magnitudes of 2, 4, 6 or 8 g/cm^2^ for 4, 16 or 24 h [10,48,49,50]. A continuous supply of oxygen under pressure application was ensured by the use of the lumox^®^ gas-permeable cell culture plates.

The proinflammatory cytokine IL-1β was applied to create an inflammatory environment in vitro. To achieve comparability of results, the same concentrations of 0.1, 1 or 10 ng/mL IL-1β as in our previous studies were used for stimulation [11,12].

In same experiment autophagy was induced pharmacologically by rapamycin (50 nM, Enzo Life Sciences, Farmingdale, NY, USA) and inhibited by chloroquine (20 µM, Enzo Life Sciences). All inhibitors were applied one hour prior to stimulation.

### 4.3. Autophagy Detection

Autophagy was measured using the Cyto-ID^®^ Autophagy Detection Kit (Enzo Life Sciences, #ENZ-51031). Cells were stimulated as described above. PDL fibroblasts were treated with 20 µM of the autophagy inhibitor chloroquine (Enzo Life Sciences, Farmingdale, NY, USA). Chloroquine disrupts the autophagic flux by inhibiting the fusion of autophagosomes with lysosomes inhibiting their degradation. Therefore, the number of autophagosomes accumulates over time accentuating the effects of the stimulation.

The staining procedure was conducted according to the manufacturer’s instructions and included a positive control with the autophagy-inducer rapamycin (offered by the kit) and a negative control (unstained cells). PDL fibroblasts were stimulated mechanically with 2, 4, 6 or 8 g/cm^2^ or under inflammatory conditions with 0.1, 1 or 10 ng/mL IL-1β for different time periods. Untreated cells served as controls. Cells were washed, collected by trypsinization and incubated with the Cyto-ID^®^ Green Detection Reagent (Enzo Life Sciences) for 30 min in the dark at room temperature. The green detection reagent accumulates specifically in autophagic vacuoles in a pH-dependent manner. Flow cytometry was performed with the FITC Filter (530/30 green) of the blue laser of a BD FACSCalibur (BD Biosciences, Franklin Lakes, NJ, USA) and analyzed with the freely available Flowing Software (http://flowingsoftware.btk.fi/).

### 4.4. Antibody Array

A human mTOR Signaling Phospho Specific Antibody Array was used for qualitative protein phosphorylation profiling of the mTOR signaling pathway (Full Moon, BioSystem, Sunnyvale, CA, USA). The array comprises 138 phospho specific antibodies for phosphorylated and unphosphorylated proteins, connected to the mTOR pathway.

In order to determine mTOR activation, PDL fibroblasts were stimulated with physiological load (2 g/cm^2^), overload (8 g/cm^2^) or cultured under inflammatory conditions (10 ng/mL IL-1β). Untreated cells served as controls. In brief, the specific antibody array was used following the manufacturer’s protocol. Cells were lysed using a protein extraction buffer provided by Full Moon (BioSystems). Biotin-labelled proteins conjugated to antibodies were detected using streptavidin-Cy3 (Full Moon BioSystems). Afterwards, slides were scanned and analyzed by Full Moon (BioSystems). Ratio changes are indicated as signal intensity changes of the phospho-site-specific antibody as compared to signal intensity changes of the associated non-phosphorylated antibody between treated and control samples. Fold change indicates signal intensity changes between treated and untreated cells.

### 4.5. Analysis of Gene Expression

Gene expressions of collagen-1 and Ki-67, an important marker for proliferation, were analyzed by real-time PCR after 24 h of stimulation. RNA was extracted with an RNA extraction kit (RNeasy Protect Minikit, Qiagen) and 0.5 μg of total RNA was used to synthesize cDNA with the iScriptTM Select cDNA Snthesis Kit (Bio-Rad Laboratories Ltd., Hercules, CA, USA) according to the manufacturer’s protocol. Furthermore, 1 μL of cDNA was used in a 25 μL reaction mixture containing 2.5 μL QuantiTect Primer Assay (Qiagen), 12.5 μL QuantiTect SYBR Green Master Mix (Qiagen) and 9 μL of nuclease free water. Real-time quantitative polymerase chain reaction (RT-qPCR) amplifications were done with an iCycler iQ5 Detection System (Bio-Rad Laboratories). The RT-qPCR protocol included a heating phase at 95 °C for 5 min to activate the enzyme, and 40 cycles, including a denaturation step at 95 °C for 10 s and a combined annealing/extension step at 60 °C for 30 s per cycle. Data normalization was carried out with the reference gene ribosomal protein L22 (RPL22) for comparative ΔΔ-CT analysis.

### 4.6. Cell Death Detection

Cell death was quantified using the reagent PI from the Vibrant^®^ Apoptosis Assay Kit #6 (V32200, Invitrogen, Corporation, Carlsbad, CA, USA) according to the manufacturer’s instructions. The reagent PI is used for staining dead cells with red fluorescence, as PI binds tightly to the nucleic acids, which are not accessible in living cells. In short, after stimulation cells were collected by trypsinization. Additionally, dead cells in cell supernatant were included in the analysis, as dead PDL fibroblasts may detach from surfaces. Cells were collected by centrifugation, washed with 1X annexin-binding buffer and treated with PI for 15 min. Finally, cells were analyzed by flow cytometry performed with 488 nm excitation for PI (BD Biosciences, Franklin Lakes, NJ, USA).

### 4.7. Statistical Analysis

For statistic evaluation, the IBM SPSS Statistics software (v. 22, IBM SPSS, Chicago, IL, USA) was used. Quantitative data are presented as means and standard errors of the mean (SEM). All experiments were performed in triplicate and reproduced at least twice. In order to compare multiple groups with the control group, the Kruskal–Wallis test followed by the Dunnett’s tests was used. Differences between groups were considered significant at *p* < 0.05.

## Figures and Tables

**Table 1:** Ratio analysis. Table 1 shows ratio changes between treated samples and control samples. The signal intensity of the phospho-site-specific antibody is compared to the signal intensity of the associated non-phosphorylated antibody. The number in parentheses indicates phosphorylation site. The table contains only data of those ratios for which at least one value of treated samples in comparison to controls is less than or equal to 0.75 or greater than or equal to 1.5.

Antibody List and Phosphorylation Site		2 g/cm^2^ /Control	8 g/cm^2^ /Control	IL-1ß /Control
4E-BP1 (Phospho-Thr45)	4E-BP1 (Ab-45)	0.72	1.18	0.96
4E-BP1 (Phospho-Ser65)	4E-BP1 (Ab-65)	0.83	1.24	1.67
AKT (Phospho-Thr308)	AKT (Ab-308)	0.71	0.86	1.09
AKT1 (Phospho-Ser124)	AKT1 (Ab-124)	1.39	1.67	1.60
AKT1 (Phospho-Thr450)	AKT1 (Ab-450)	1.15	1.40	1.52
AKT1 (Phospho-Tyr474)	AKT1 (Ab-474)	0.98	0.73	1.03
AMPK1/AMPK2 (Phospho-Thr183/172)	AMPK1/AMPK2 (Ab-183/172)	1.08	0.74	0.99
BAD (Phospho-Ser112)	BAD (Ab-112)	0.89	0.99	0.73
BAD (Phospho-Ser91/128)	BAD (Ab-91/128)	0.90	1.63	1.23
ERK3 (Phospho-Ser189)	ERK3 (Ab-189)	1.35	1.97	1.40
GSK3α (Phospho-Ser21)	GSK3α (Ab-21)	0.82	0.76	0.70
GSK3β (Phospho-Ser9)	GSK3β (Ab-9)	0.77	0.68	0.64
Mnk1 (Phospho-Thr385)	Mnk1 (Ab-385)	1.16	1.66	1.36
P70S6K (Phospho-Thr229)	P70S6K (Ab-229)	1.53	1.25	1.54
P70S6K (Phospho-Ser418)	P70S6K (Ab-418)	1.99	2.21	2.33
P70S6K beta (Phospho-Ser423)	P70S6K beta (Ab-423)	1.30	1.60	1.18
P90RSK (Phospho-Thr359/Ser363)	P90RSK (Ab-359/363)	2.23	1.54	2.36
PI3-kinase p85-subunit alpha/gamma (Phospho-Tyr467/Tyr199)	PI3-kinase p85-subunit alpha/gamma (Ab-467/199)	1.14	1.70	1.60
PP2A-alpha (Phospho-Tyr307)	PP2A-alpha (Ab-307)	1.32	1.17	1.90
PPAR-gamma (Phospho-Ser112)	PPAR-gamma (Ab-112)	1.43	2.08	2.43
PTEN (Phospho-Ser370)	PTEN (Ab-370)	1.91	1.50	2.05
PTEN (Phospho-Ser380)	PTEN (Ab-380)	0.83	0.97	1.67
PTEN (Phospho-Ser380/Thr382/Thr383)	PTEN (Ab-380/382/383)	1.51	2.02	1.47
Rho/Rac guanine nucleotide exchange factor 2 (Phospho-Ser885)	Rho/Rac guanine nucleotide exchange factor 2 (Ab-885)	0.99	1.66	1.13
RSK1/2/3/4 (Phospho-Ser221/227/218/232)	RSK1/2/3/4 (Ab-221/227/218/232)	1.24	1.52	1.36
Tuberin/TSC2 (Phospho-Thr1462)	Tuberin/TSC2 (Ab-1462)	1.08	0.70	0.92
Tuberin/TSC2 (Phospho-Ser939)	Tuberin/TSC2 (Ab-939)	1.27	0.73	1.20

**Table 2:** Fold changes between treatment samples in comparison to controls. This table contains a selection of interesting targets associated with the autophagy regulation pathway mTOR. The table contains only fold changes of those targets where at least one value of treated samples in comparison to controls is less than or equal to 0.75 or greater than or equal to 1.5. The complete dataset of fold changes can be found in Supplement 1.

Antibody List	2 g/cm^2^ /Control	8 g/cm^2^ /Control	IL-1β /Control
4E-BP1 (Ab-65)	1.34	0.94	0.65
4E-BP1 (Phospho-Thr45)	0.61	1.12	0.81
AKT (Ab-308)	0.85	0.68	0.71
AKT (Ab-473)	0.88	0.69	0.87
AKT (Phospho-Thr308	0.60	0.58	0.77
AKT1 (Ab-124)	0.78	0.73	0.73
AKT1 (Phospho-Tyr474)	0.80	0.71	0.82
AKT1S1 (Ab-246)	0.81	0.88	0.69
AKT2 (Phospho-Ser474)	0.79	0.90	0.72
mTOR (Ab-2446)	0.90	0.71	0.93
mTOR (Ab-2481)	0.90	1.02	0.67
mTOR (Phospho-Ser2481)	0.89	0.79	0.75
mTOR (Phospho-Thr2446)	0.75	1.00	0.93
P70S6K (Ab-229)	0.77	0.95	0.74
P70S6K (Ab-418)	0.85	0.86	0.74
P70S6K (Phospho-Thr389)	1.35	1.53	1.28
P70S6K beta (Ab-423)	0.87	0.68	0.81
PI3-kinase p85-subunit alpha/gamma (Ab-467/199)	0.95	0.65	0.62
PTEN (Ab-370)	0.64	0.73	0.55
PTEN (Ab-380)	1.14	0.99	0.63
PTEN (Ab-380/382/383)	0.78	0.71	0.75
P70S6K (Phospho-Thr389)	1.35	1.53	1.28

## Supplementary Materials

Supplementary materials can be found at https://www.mdpi.com/1422-0067/21/24/9446/s1. Table S1: Fold changes between samples. Table S1 shows fold changes between treatment samples in comparison to controls. Table S1 contains all investigated targets associated to the autophagy regulation pathway mTOR.
