# Supplementary material for: Regulation of Autophagic Signaling by Mechanical Loading and Inflammation in Human PDL Fibroblasts
Source: Int J Mol Sci. 2020 Dec 11;21(24):9446. doi: 10.3390/ijms21249446 (PMC7763506; doi:10.3390/ijms21249446)
Supplement: Supplementary file 1 [file ijms-21-09446-s001.pdf]

International Journal of *Molecular Sciences*

MDPI

Supplementary Materials

Table S1. Fold changes between samples. Table S1 shows fold changes between treatment samples in comparison to controls. Table S1 contains all investigated targets associated to the autophagy regulation pathway mTOR.

Antibody List	2 g/cm²/Control	8 g/cm²/Control	1l-1β/Control
4E-BP1 (Ab-36)	0.92	0.86	0.86
4E-BP1 (Ab-45)	0.84	0.95	0.85
4E-BP1 (Ab-65)	1.34	0.94	0.65
4E-BP1 (Ab-70)	0.95	0.87	0.93
4E-BP1 (Phospho-Ser65)	1.12	1.16	1.09
4E-BP1 (Phospho-Thr36)	1.01	1.01	1.02
4E-BP1 (Phospho-Thr45)	0.61	1.12	0.81
4E-BP1 (Phospho-Thr70)	0.98	1.14	1.02
6-Phosphofructo-2-Kinase (PFKFB2) (inter)	0.84	0.84	0.85
6-phosphofructo-2-kinase/fructose-2.6-biphosph atase 2 (PFKFB2) (Ab-483)	1.15	1.26	1.03
6-phosphofructo-2-kinase/fructose-2.6-biphosph atase 2 (PFKFB2) (Phospho-Ser483)	0.92	1.01	0.84
AKT (Ab-308)	0.85	0.68	0.71
AKT (Ab-326)	1.07	1.45	1.03
AKT (Ab-473)	0.88	0.69	0.87
AKT (Phospho-Ser473)	1.01	0.91	1.03
AKT (Phospho-Thr308)	0.60	0.58	0.77
AKT (Phospho-Tyr326)	1.01	1.17	0.93
AKT1 (Ab-124)	0.78	0.73	0.73
AKT1 (Ab-246)	0.88	1.02	0.87
AKT1 (Ab-450)	0.97	0.77	0.82
AKT1 (Ab-474)	0.81	0.98	0.80
AKT1 (Ab-72)	0.91	1.08	0.86
AKT1 (Phospho-Ser124)	1.08	1.22	1.17
AKT1 (Phospho-Ser246)	1.08	1.03	0.98
AKT1 (Phospho-Thr450)	1.11	1.08	1.24
AKT1 (Phospho-Thr72)	0.93	0.85	0.92
AKT1 (Phospho-Tyr474)	0.80	0.71	0.82
AKT1S1 (Ab-246)	0.81	0.88	0.69
AKT1S1 (Phospho-Thr246)	0.91	0.94	0.89
AKT2 (Ab-474)	1.00	0.84	0.78
AKT2 (Phospho-Ser474)	0.79	0.90	0.72
AMPK1/AMPK2 (Ab-183/172)	0.95	1.07	0.97
AMPK1/AMPK2 (Ab-485/491)	1.03	0.79	0.96
AMPK1/AMPK2 (Phospho-Ser485/491)	1.06	1.02	1.00
AMPK1/AMPK2 (Phospho-Thr183/172)	1.09	1.12	0.83
AMPKbeta1 (Ab-182)	1.04	0.97	0.91
AMPKbeta1 (Phospho-Ser182)	1.13	1.25	1.24
BAD (Ab-112)	1.00	0.86	0.88
BAD (Ab-134)	0.90	0.75	0.89
BAD (Ab-136)	1.00	0.77	0.88
BAD (Ab-155)	1.03	0.87	0.94
BAD (Ab-91/128)	1.13	0.74	0.87
BAD (Phospho-Ser112)	0.89	0.85	0.65
BAD (Phospho-Ser134)	0.95	0.98	0.79
BAD (Phospho-Ser136)	0.81	0.79	0.76

BAD (Phospho-Ser155)	0.97	1.04	0.95
BAD (Phospho-Ser91/128)	1.01	1.20	1.07
Beta actin	1.08	0.94	0.80
CBP (inter)	0.88	0.82	0.80
CBPN (inter)	0.82	0.86	0.83
eIF2A (Ab-51)	0.97	0.95	0.86
eIF2A (Phospho-Ser51	1.01	1.05	0.98
eIF4B (Phospho-Ser422)	0.99	1.13	1.02
eIF4E (Ab-209)	0.86	0.93	0.95
eIF4E (Phospho-Ser209)	0.94	1.07	0.95
eIF4G (Ab-1108)	0.96	1.12	0.77
eIF4G (Phospho-Ser1108)	1.24	1.10	0.91
ERK1/2 (N-term)	0.69	0.61	0.61
ERK1-p44/42 MAP Kinase (Ab-202)	0.86	0.91	0.89
ERK1-p44/42 MAP Kinase (Ab-204)	0.80	0.90	0.83
ERK1-p44/42 MAP Kinase (Phospho-Thr202)	0.91	0.91	0.83
ERK1-p44/42 MAP Kinase (Phospho-Tyr204)	0.95	0.97	1.01
ERK3 (Ab-189)	0.83	0.65	0.81
ERK3 (Phospho-Ser189)	1.13	1.28	1.13
ERK8 (Phospho-Thr175/Tyr177)	0.99	1.17	1.06
GAPDH	1.06	1.08	1.16
GSK3α (Ab-21)	0.96	0.95	0.89
GSK3α (Phospho-Ser21)	0.78	0.73	0.63
GSK3α-β (Ab-216/279)	0.90	0.69	0.88
GSK3α-β (Phospho-Tyr216/279)	0.94	0.87	0.83
GSK3β (Ab-9)	1.32	1.36	1.27
GSK3β (Phospho-Ser9)	1.01	0.93	0.81
IR (Phospho-Tyr1355)	1.05	0.81	1.12
IR (Phospho-Tyr1361)	1.26	0.67	0.99
LRP11 (inter)	0.90	0.95	0.88
LRP3 (inter)	1.01	1.12	1.07
Mnk1 (Ab-385)	0.85	0.83	0.89
Mnk1 (Phospho-Thr385)	0.99	1.38	1.21
mTOR (Ab-2446)	0.90	0.71	0.93
mTOR (Ab-2448)	1.07	0.87	0.83
mTOR (Ab-2481)	0.90	1.02	0.67
mTOR (Phospho-Ser2448)	0.91	1.13	0.93
mTOR (Phospho-Ser2481)	0.89	0.79	0.75
mTOR (Phospho-Thr2446)	0.75	1.00	0.93
P70S6K (Ab-229)	0.77	0.95	0.74
P70S6K (Ab-371)	1.10	1.19	1.08
P70S6K (Ab-411)	0.97	0.88	0.80
P70S6K (Ab-418)	0.85	0.86	0.74
P70S6K (Ab-421)	1.02	0.77	0.96
P70S6K (Ab-424)	0.91	0.94	0.83
P70S6K (Ab-427)	0.91	0.68	0.85
P70S6K (Phospho-Ser371)	1.27	1.38	1.31
P70S6K (Phospho-Ser411)	0.96	1.10	1.03
P70S6K (Phospho-Ser418)	1.69	1.90	1.73
P70S6K (Phospho-Ser424)	1.03	1.14	1.04
P70S6K (Phospho-Thr229)	1.18	1.19	1.14
P70S6K (Phospho-Thr389)	1.35	1.53	1.28
P70S6K (Phospho-Thr421)	0.96	0.94	0.92
P70S6K beta (Ab-423)	0.87	0.68	0.81
P70S6K beta (Phospho-Ser423)	1.12	1.09	0.96
P90RSK (Ab-359/363)	0.80	0.61	0.51

P90RSK (Ab-380)	0.89	0.86	0.82
P90RSK (Ab-573)	0.95	0.92	0.88
P90RSK (Phospho-Ser380)	1.09	0.92	1.12
P90RSK (Phospho-Thr359/Ser363)	1.79	0.94	1.21
P90RSK (Phospho-Thr573)	1.18	1.04	1.29
PDK1 (Ab-241)	1.16	1.21	0.77
PDK1 (Phospho-Ser241)	0.93	0.95	0.77
PI3-kinase p85-alpha (Phospho-Tyr607)	1.02	1.10	1.20
PI3-kinase p85-subunit alpha/gamma (Ab-467/199)	0.95	0.65	0.62
PI3-kinase p85-subunit alpha/gamma (Phospho-Tyr467/Tyr199)	1.08	1.10	0.99
PIP5K (inter)	1.01	0.99	1.26
PIP5K (Phospho-Ser307)	1.33	1.58	1.14
PKC alpha (Ab-657)	1.36	1.52	1.28
PKC alpha (Phospho-Tyr657)	1.17	1.57	1.29
PKC alpha/beta II (Ab-638)	0.94	1.20	0.91
PKC alpha/beta II (Phospho-Thr638)	1.09	1.06	1.08
PLK1 (Ab-210)	0.57	0.53	0.50
PP2A-alpha (Ab-307)	0.84	0.82	0.59
PP2A-alpha (Phospho-Tyr307)	1.11	0.96	1.12
PPAR-beta (Ab-1457)	0.86	0.72	0.75
PPAR-beta (Phospho-Thr1457)	0.97	1.04	0.96
PPAR-gamma (Ab-112)	0.88	0.64	0.46
PPAR-gamma (Phospho-Ser112)	1.26	1.33	1.12
PTEN (Ab-370)	0.64	0.73	0.55
PTEN (Ab-380)	1.14	0.99	0.63
PTEN (Ab-380/382/383)	0.78	0.71	0.75
PTEN (Phospho-Ser370)	1.23	1.10	1.14
PTEN (Phospho-Ser380)	0.94	0.96	1.05
PTEN (Phospho-Ser380/Thr382/Thr383)	1.18	1.43	1.10
Rho/Rac guanine nucleotide exchange factor 2 (Ab-885)	0.95	0.71	0.90
Rho/Rac guanine nucleotide exchange factor 2 (Phospho-Ser885)	0.94	1.17	1.02
RhoA (Ab-188)	0.93	0.90	0.83
RSK1/2/3/4 (Ab-221/227/218/232)	0.89	0.84	0.72
RSK1/2/3/4 (Phospho-Ser221/227/218/232)	1.10	1.27	0.99
Tuberin (Ab-981)	0.57	0.46	0.54
Tuberin/TSC2 (Ab-1462)	0.92	1.04	0.96
Tuberin/TSC2 (Ab-939)	0.86	1.04	0.83
Tuberin/TSC2 (Phospho-Ser939)	1.09	0.76	1.00
Tuberin/TSC2 (Phospho-Thr1462)	0.99	0.73	0.88

References

1. Alqurashi, N.; Hashimi, S.M.; Alowaidi, F.; Ivanovski, S.; Wei, M.Q. Dual mTOR/PI3K inhibitor NVP-BEZ235 arrests colorectal cancer cell growth and displays differential inhibition of 4E-BP1. *Oncol. Rep.* **2018**, *40*, 1083–1092.
2. Antonioli, M.; Di Rienzo, M.; Piacentini, M.; Fimia, G.M. Emerging Mechanisms in Initiating and Terminating Autophagy. *Trends Biochem. Sci.* **2017**, *42*, 28–41.
3. Araujo, R.M.S.; Oba, Y.; Moriyama K. Identification of Genes Related to Mechanical Stress in Human Periodontal Ligament Cells Using Microarray Analysis. *J. Periodontal Res.* **2007**, *42*, 15–22.
4. De Araujo, R.M.S.; Oba, Y.; Kuroda, S.; Tanaka, E.; Moriyama, K. RhoE regulates actin cytoskeleton organization in human periodontal ligament cells under mechanical stress. *Arch. Oral Biol.* **2014**, *59*, 187–192.
5. Bakker, A.D.; Gakes, T.; Hogervorst, J.M.A.; de Wit, G.M.J.; Klein-Nulend, J.; Jaspers, R.T. Mechanical Stimulation and IGF-1 Enhance mRNA Translation Rate in Osteoblasts Via Activation of the AKT-mTOR Pathway. *J Cell Physiol.* **2016**, *231*, 1283–1290.
6. Barczyk, M.; Bolstad A.I.; Gullberg, D. Role of integrins in the periodontal ligament: organizers and facilitators. *Periodontol. 2000.* **2013**, *63*, 29–47. doi: 10.1111/prd.12027.
7. Chen, Y.; Yang, K.; Zhou, Z.; Wang, L.; Du, Y.; Wang, X. Mechanical Stress Modulates the RANKL/OPG System of Periodontal Ligament Stem Cells via α 7 nAChR in Human Deciduous Teeth: An In Vitro Study. *Stem Cells Int.* **2019**, 1–12.
8. Chen, H.; Chen, L.; Cheng, B.; Jiang, C. Cyclic mechanical stretching induces autophagic cell death in tenofibroblasts through activation of prostaglandin E2 production. *Cell Physiol. Biochem*. **2015**, *36*, 24–33.
9. Cominelli, F.; Nast, C.C.; Llerena, R.; Dinarello, C.A.; Zipser, R.D. Interleukin 1 suppresses inflammation in rabbit colitis. Mediation by endogenous prostaglandins. *J. Clin. Invest.* **1990**, *85*, 582–586.
10. Ekim, B.; Magnuson, B.; Acosta-Jaquez, H.A.; Keller, J.A.; Feener, E.P.; Fingar, D.C. mTOR Kinase Domain Phosphorylation Promotes mTORC1 Signaling. Cell Growth. and Cell Cycle Progression. *Mol. Cell. Biol.* **2011**, *31*, 2787–2801.
11. Hannan, K.M.; Brandenburger, Y.; Jenkins, A.; Sharkey, K.; Cavanaugh, A.; Rothblum, L.; Moss, T.; Poortinga, G.; McArthur, G.A.; Pearson, R.B.; Hannan, R.D. mTOR-dependent regulation of ribosomal gene transcription requires S6K1 and is mediated by phosphorylation of the carboxy-terminal activation domain of the nucleolar transcription factor UBF. *Mol. Cell. Biol.* **2003**, *23*, 8862–8877.
12. Harris, J. Autophagy and cytokines. *Cytokine* **2011**, *56*, 140–144.
13. Huang, W.-P.; Klionsky. D.J. Autophagy in yeast: a review of the molecular machinery. *Cell Struct. Funct.* **2002**, *27*, 409-420.
14. Hudson, D.M.; Garibov, M.; Dixon, D.R.; Popowics, T.; Eyre, D.R. Distinct post-translational features of type I collagen are conserved in mouse and human periodontal ligament. *J. Periodontal Res.* **2017**, *52*, 1042–1049.
15. Kaku, M.; Yamauchi, M. Mechano-regulation of collagen biosynthesis in periodontal ligament. *J Prosthodont. Res.* **2014**, *58*, 193–207.
16. Kanzaki, H.; Chiba, M.; Shimizu, Y.; Mitani, H. Periodontal ligament cells under mechanical stress induce osteoclastogenesis by receptor activator of nuclear factor kappaB ligand up-regulation via prostaglandin E2 synthesis. *J. Bone Miner. Res*. **2002**, *17*, 210–220.
17. King, J.S.; Veltman, D.M.; Insall, R.H. The induction of autophagy by mechanical stress. *Autophagy* **2011**, *7*, 1490–1499.
18. King, J.S. Mechanical stress meets autophagy: potential implications for physiology and pathology. *Trends Mol. Med.* **2012**, *18*, 583–588.
19. Kirschneck, C.; Küchler, E.C.; Wolf, M.; Spanier, G.; Proff, P.; Schröder, A. Effects of the Highly COX-2-Selective Analgesic NSAID Etoricoxib on Human Periodontal Ligament Fibroblasts during Compressive Orthodontic Mechanical Strain. *Mediators Inflamm.* **2019**, *2019*, 2514956.
20. Klionsky, D.J.; Abdelmohsen, K.; Abe, A.; Abedin, M.J.; Abeliovich, H.; Arozena, A.A.; Adachi, H.; Adams, C.M.; Adams, P.D.; Adeli, K.; et al. Guidelines for the use and interpretation of assays for monitoring autophagy (3rd edition). *Autophagy* **2016**, *12*, 1–222.
21. Lapaquette, P.; Guzzo, J.; Bretillon, L.; Bringer, M.-A. Cellular and Molecular Connections between Autophagy and Inflammation. *Mediators Inflamm.* **2015**, *2015*, 398483.

22. Lee, K.-W.; Yook, J.-Y.; Son, M.-Y.; Kim, M.-J.; Koo, D.-B.; Han, Y.-M.; Cho, Y.S. Rapamycin Promotes the Osteoblastic Differentiation of Human Embryonic Stem Cells by Blocking the mTOR Pathway and Stimulating the BMP/Smad Pathway. *Stem Cell. Dev.* **2010**, *19*, 557–568.
23. Li, L.; Jacox, L.A.; Little, S.H.; Ko, C.-C. Orthodontic tooth movement: The biology and clinical implications. *Kaohsing J. Med. Sci.* **2018**, *34*, 207–214.
24. Li, Y.; Li, M.; Tan, L.; Huang, S.; Zhao, L.; Tang, T.; Liu, J.; Zhao, Z. Analysis of time-course gene expression profiles of a periodontal ligament tissue model under compression. *Arch. Oral Biol.* **2013**, *58*, 511–522.
25. Ma, K.-G.; Shao, Z.-W.; Wang, J.; Wang, B.-C.; Xiong, L.-M.; Chen, S.-F. Autophagy is activated in compression-induced cell degeneration and is mediated by reactive oxygen species in nucleus pulposus cells exposed to compression. *Osteoarthritis Cartilage* **2013**, *21*, 2030–2038.
26. Mangan, D.F.; Welch, G.R.; Wahl, S.M. Lipopolysaccharide. tumor necrosis factor-alpha. and IL-1 beta prevent programmed cell death (apoptosis) in human peripheral blood monocytes. *J. Immunol.* **1991**, *146*, 1541–1546/
27. Marchesan, J.T.; Scanlon, C.S.; Soehren, S.; Matsuo, M.; Kapila, Y.L. Implications of cultured periodontal ligament cells for the clinical and experimental setting: a review. *Arch. Oral Biol.* **2011**, *56*, 933–943.
28. Mariño, G.; Niso-Santano, M.; Baehrecke, E.H.; Kroemer, G. Self-consumption: the interplay of autophagy and apoptosis. *Nat. Rev. Mol. Cell Biol.* **2014**, *15*, 81–94.
29. Memmert, S.; Damanaki, A.; Nogueira, A.V.B.; Eick, S.; Nokhbehsaim, M.; Papadopoulou, A.K.; Till, A.; rath, B.; Jepsen, S.; Götz, W.; Piperi, C.; et al. Role of Cathepsin S in Periodontal Inflammation and Infection. *Mediators Inflamm.* **2017**, *2017*, 4786170.
30. Memmert, S.; Nogueira, A.V.B.; Damanaki, A.; Nokhbehsaim, M.; Eick, S.; Divnic-Resnik, T.; Spahr, A.; rath-Deschner, B.; Till, A.; Götz, W.; et al. Damage-regulated autophagy modulator 1 in oral inflammation and infection. *Clin. Oral Investig.* **2018**, *22*, 2933–2941, doi: 10.1007/s00784-018-2381-6.
31. Memmert, S.; Damanaki, A.; Nohbehsaim, M.; Nogueira, A.V.B.; Eick, S.; Cirelli, J.A.; Jäger, J. Regulation of somatostatin receptor 2 by proinflammatory. microbial and obesity-related signals in periodontal cells and tissues. *Head Face Med.* **2019**, *15*, 2
32. Memmert, S.; Nogueira, A.V.B.; Damanaki, A.; Nokhbehsaim, M.; Rath-Deschner, B.; Götz, W; Götz, L.; Cirelli, J.A.; Till, A; Jäger, J.; et al. Regulation of the autophagy-marker Sequestosome 1 in periodontal cells and tissues by biomechanical loading. *J. Orofac. Orthop*. **2020**, *81*, 10–21.
33. Militi, A.; Cutroneo, G.; Favaloro, A.; Matarese, G.; Di Mauro, D.; Lauritano, F.; Centofanti, A.; Cervino, G.; Nicita, F.; Bramanti, A.; et al.An immunofluorescence study on VEGF and extracellular matrix proteins in human periodontal ligament during tooth movement. *Heliyon* **2019**, *5*, e02572.
34. Muendlein, H.I.; Sarhan, J.; Liu, B.C.; Connolly, W.M.; Schworer, S.A.; Smirnova, I.; Tang, A.Y.; Ilyukha, V.; Pietruska, J.; Tahmasebi, S.; et al. Constitutive Interferon Attenuates RIPK1/3-Mediated Cytokine Translation. *Cell Rep.* **2020**, *30*, 699–713.
35. Nakajima, R.; Yamaguchi, M.; Kojima, T.; Takano, M.; Kasai, K. Effects of compression force on fibroblast growth factor-2 and receptor activator of nuclear factor kappa B ligand production by periodontal ligament cells in vitro. *J. Periodontal Res.* **2008**, *43*, 168–173.
36. Pietrocola, F.; Izzo, V.; Niso-Santano, M.; Vacchelli, E.; Galluzzi, L.; Maiuri, M.C.; Kroemer, G. Regulation of autophagy by stress-responsive transcription factors. *Semin Cancer Biol.* **2013**, *23*, 310–322.
37. Reiling, J.H.; Sabatini, D.M. Stress and mTORture signaling. *Oncogene* **2006**, *25*, 6373–6383.
38. Schröder, A.; Bauer, K.; Spanier, G.; Proff, P.; Wolf, M.; Kirschneck, C. Expression kinetics of human periodontal ligament fibroblasts in the early phases of orthodontic tooth movement. *J. Orofac. Orthop.* **2018**, *79*, 337–351.
39. Seveau, S.; Turner, J.; Gavrilin, M.A.; Torrelles, J.B.; Hall-Stoodley, L.; Yount, J.S.; Amer, A.O. Checks and Balances between Autophagy and Inflammasomes during Infection. *J. Mol. Biol.* **2018**, *430*, 174–192.
40. Song, F.; Wang, Y.; Jiang, D.; Wang, T.; Zhang, Y.; Ma, H.; Kang, Y. Cyclic Compressive Stress Regulates Apoptosis in Rat Osteoblasts: Involvement of PI3K/Akt and JNK MAPK Signaling Pathways. *PLoS One* **2016**, *11*, e0165845.
41. Song, F.; Jiang, D.; Wang, Y.; Lou, Y.; Zhang, Y.; Kang, Y. Mechanical Stress Regulates Osteogenesis and Adipogenesis of Rat Mesenchymal Stem Cells through PI3K/Akt/GSK-3 β/β -Catenin Signaling Pathway. *BioMed Res. Int*. **2017**, 1–10.

42. Tang, L.-L.; Xian, C.-Y.; Wang, Y.-L. The MGF expression of osteoblasts in response to mechanical overload. *Arch. Oral Biol.* **2006**, *51*, 1080–1085.
43. Uchiyama, Y.; Shibata, M.; Koike, M.; Yoshimura, K.; Sasaki, M. Autophagy–physiology and pathophysiology. *Histochem. Cell Biol.* **2008**, *129*, 407–420.
44. Ullrich, N.; Schroder, A.; Jantsch, J.; Spanier, G.; Proof, P.; Kirschneck, C. The role of mechanotransduction versus hypoxia during simulated orthodontic compressive strain-an in vitro study of human periodontal ligament fibroblasts. *Int. J. Oral Sci.* **2019**, *11*, 33.
45. Vercammen, E.; Staal, J.; Van Den Broeke, A.; Haegman, M.; Vereecke, L.; Schotte, P.; Bayaert, R. Prolonged exposure to IL-1b and IFNc induces necrosis of L929 tumor cells via a p38MAPK/NF-jB/NO-dependent mechanism. *Oncogene* **2008**, *27*, 3780–3788.
46. Wang, H.; Liu, Y.; Wang, D.; Xu, Y.; Dong, R.; Yang, Y.; Lv, Q.; Chen, X.; Zhang, Z. The Upstream Pathway of mTOR-Mediated Autophagy in Liver Diseases. *Cells* **2019**, *8*, 1597.
47. Wolf, M.; Lossdorfer, S.; Craveiro, R.; Gotz, W.; Jager, A. Regulation of macrophage migration and activity by high-mobility group box 1 protein released from periodontal ligament cells during orthodontically induced periodontal repair: an in vitro and in vivo experimental study. *J. Orofac. Orthop.* **2013**, *74*, 420–434.
48. Wu, Y.; Zhao, D.; Zhuang, J.; Zhang, F.; Xu, C. Caspase-8 and Caspase-9 Functioned Differently at Different Stages of the Cyclic Stretch-Induced Apoptosis in Human Periodontal Ligament Cells. *PLoS One* **2016**, *11*, e0168268.
49. Xiang, X.; Zhao, J.; Xu, G.; Li, Y.; Zhang, W. mTOR and the differentiation of mesenchymal stem cells. *Acta Biochim. Biophys. Sin. (Shanghai)* **2011**, *43*, 501–510.
50. Young, M.M.; Takahashi, Y.; Kahn, O.; Park, S.; Hori, T.; Yun, J.; Sharma, A.K.; Amin, S.; Hu, C.-D.; Zhang, J.; et al. Autophagosomal membrane serves as platform for intracellular death-inducing signaling complex (iDISC)-mediated caspase-8 activation and apoptosis. *J. Biol. Chem.* **2012**, *287*, 12455–12468.
51. Yang, L.; Miao, L.; Liang, F.; Huang, H.; Teng, X.; Li, S.; Nuriddinov, J.; Selzer, M.E.; Hu, Y. The mTORC1 effectors S6K1 and 4E-BP play different roles in CNS axon regeneration. *Nat. Commun.* **2014.**
52. Zhang, X.; Kohli, M.; Zhou, Q.; Graves, D.T.; Amar, S. Short- and Long-Term Effects of IL-1 and TNF Antagonists on Periodontal Wound Healing. *J Immunol.* **2004**, *173*, 3514–3523.
